# *Leishmania braziliensis* SCD6 and RBP42 proteins, two factors with RNA binding capacity

**DOI:** 10.1186/s13071-017-2557-y

**Published:** 2017-12-19

**Authors:** Paola A. Nocua, Cesar A. Ramirez, José M. Requena, Concepción J. Puerta

**Affiliations:** 1 Laboratorio de Parasitología Molecular, Facultad de Ciencias, Pontificia Universidad Javeriana, Bogotá, Colombia; 2 Centro de Biología Molecular Severo Ochoa (CSIC-UAM), Universidad Autónoma de Madrid, Madrid, Spain

**Keywords:** *Leishmania braziliensis*, RNA-binding proteins, NTF2 domain, Lsm domain, RRM domain, Heat shock

## Abstract

**Background:**

The study of RNA binding proteins (RBPs) is of great relevance for understanding processes like post-transcriptional control of gene expression. The post-transcriptional mechanisms are particularly important in *Leishmania* parasites and related trypanosomatids since transcriptional regulation is almost absent in them. Thus, RBPs should be essential during the development of these parasites and for survival strategies against the adverse conditions that they face during their life-cycle. This work was aimed to do a structural and biochemical characterization of two *Leishmania braziliensis* proteins, which were previously found in pull-down assays using an *HSP70* RNA as bait. At that time, these proteins were annotated as hypothetical proteins (LbrM.25.2210 and LbrM.30.3080) in the GeneDB database.

**Results:**

Structural analysis indicated that these two proteins belong to evolutionarily conserved families; thus, they have been renamed accordingly as LbSCD6 (LbrM.25.2210) and LbRBP42 (LbrM.30.3080). We have demonstrated experimentally that these proteins are RBPs, in agreement with their structural features. Both proteins were able to bind to the complete 3′ UTR-II region of HSP70-type II mRNA, and to an A + U rich element (ARE) present in that UTR. Cellular localization assays suggested that both proteins are mainly distributed in the cytoplasm of promastigotes growing at 26 °C, but they accumulate in foci around the nucleus when the parasites are under heat-shock conditions. Also, our study showed that steady-state levels of LbSCD6 and LbRBP42 transcripts decreased significantly during incubation of *L. braziliensis* promastigotes at heat-shock temperatures. However, in these conditions, the cellular content of both proteins remained unaltered.

**Conclusions:**

Our data suggest that LbSCD6 and LbRBP42, as occurs for their orthologues in other organisms, are involved in mRNA regulation, and probably they have a relevant role facing the stress conditions that *L. braziliensis* encounters during insect-to-mammalian transmission.

**Electronic supplementary material:**

The online version of this article (10.1186/s13071-017-2557-y) contains supplementary material, which is available to authorized users.

## Background

*Leishmania* are protozoan parasites that cause leishmaniasis, a broad spectrum of pathological outcomes in humans, ranging from self-healing cutaneous lesions to mucocutaneous affections, with extensive tissue destruction, and to fatal visceral disease. In immunocompetent persons, the severity of the clinical manifestations depends mainly on the particular infecting species; thus, for example, mucocutaneous leishmaniasis is a hallmark of *L. braziliensis* infection [1]. These parasites present a complex digenetic life-cycle alternating between invertebrate and vertebrate hosts. Therefore, these parasites must develop morphological and biochemical changes that allow them to survive the host defense mechanisms and adapt to the intracellular environment within the human phagocytic cells [2]. Consequently, an accurate regulation of parasite gene expression is essential to direct the differentiation from the promastigote form in sand flies to the amastigote inside the mammalian cells, and vice versa [3].

Unlike most eukaryotic cells, gene regulation in these parasites occurs almost exclusively at the post-transcriptional level, which could be a consequence of their unusual chromosomal arrangement; *Leishmania* genes are ordered in large clusters comprising up to hundreds of genes with the same transcriptional orientation [4]. For these parasites, no RNA polymerase II promoters, driving expression of protein coding, have been described. Moreover, transcription starts at divergent strand switch regions (SSR) and proceeds bi-directionally towards either the chromosomal telomeres or convergent SSR in which transcription ends [5]. As a result, there is a constitutive expression of most chromosomal regions that are transcribed into polycistronic RNA precursors, which are ultimately processed as individual mRNAs. Maturation of RNAs involves the addition, by *trans*-splicing, of a common miniexon at the 5′-end and a poly(A) tail at the 3′, by polyadenylation; these two reactions are temporal and spatially coupled [6]. Therefore, the differential expression of mRNAs in *Leishmania*, and related trypanosomatids, is attained via regulation of their processing, transport, stability and translation [7, 8]. It is conceivable that these processes are controlled by the interaction with different RNA-binding proteins (RBPs), which would recognize their mRNA targets via *cis*-acting elements, mainly located on the 5’and 3′-untranslated regions [7, 9]. Hence, the study of the *trans*-acting factors and *cis*-regulatory sequences involved in these regulatory mechanisms are of great importance.

In a recent study, our group identified 52 different proteins associated with the 5′ and 3′ UTRs of the mRNAs coding for the *L. braziliensis* 70-kDa heat-shock protein (*HSP70)* [10]. Eighteen of the identified proteins corresponded to proteins known to be involved in cellular processes related to RNA metabolism and translation. Another five proteins were annotated in the GeneDB database as hypothetical conserved proteins; two of them recalled our attention due to the presence in their structures of motifs with potential for interaction with RNA. They correspond to the proteins encoded by the annotated genes *LbrM.25.2210* and *LbrM.30.3080*. Particularly, these proteins were identified because of their association with the 3′ UTR of the *HSP70-II* gene in extracts from parasites grown at 26 °C. This feature was of particular interest since it had been reported that transcripts derived from the *HSP70-II* genes at 26 °C are stored in *Leishmania* parasites to provide de novo synthesis of HSP70 when parasites face stress conditions [11]. Therefore, we undertook the objective of characterizing these two proteins, considering that they may be playing a relevant role in the regulation of *HSP70* mRNAs. Preliminary bioinformatic analysis showed that the protein, encoded by the gene *LbrM.25.2210*, presents motifs common to members of the Sm-like protein family. Proteins of this family in *Drosophila* (TraI), nematodes (CAR-I), yeasts (Scd6), plants (DCP5), and humans (RAP55) have been involved in translational repression and accumulation of mRNAs into P bodies and stress granules [12–16]. Here, in order to reflect its evolutionary origin, the protein was named LbSCD6. On the other hand, the protein encoded by *LbrM.30.3080* gene (renamed here LbRBP42) was found to present the structural NTF2-like motif, which is typical of proteins of the nuclear transport factor 2 like protein family. Members of this family have been involved in nucleoplasmic transport and RNA export processes [17]. Recently, its homologue in *Trypanosoma brucei*, the TbRPB42 protein, has been found to be essential and a preferential interaction with mRNAs encoding proteins involved in cellular energy metabolism in the insect form of this parasite was suggested [18].

In addition to the structural characterization of the LbSCD6 and LbRBP42 proteins in this study, we have analyzed their RNA-binding capacities*.* In particular, we addressed the ability of the recombinant proteins to interact with the 3′ UTR of *L. braziliensis* HSP70-II mRNAs. Finally, to provide clues about their functional activity in vivo, we determined their mRNA and protein expression levels as well as their cellular location under normal and heat-shock conditions.

## Methods

### Bioinformatics analysis of LbSCD6 and LbRBP42 proteins

Multiple alignments of sequences of each protein with its respective homologous were carried out using ClustalW. Domain identification was done using the InterProScan tool [19].

Molecular modeling was done using the Phyre2 server [20] using a protein-threading method, as there are not experimentally solved structures for orthologous proteins. The stereochemical quality of the modeled protein structures were validated by the PROCHECK program [21], available at EBI server. The protein models were visualized with the program MacPyMOL Molecular Graphics System, Version 1.7.2.1.

### Parasite cultures, nucleic acid and proteins extraction

The *L. braziliensis* MHOM/BR/75/M2904 strain was provided by CIDEIM (Centro Internacional de Entrenamiento e Investigaciones Médicas, Colombia) on June 2008. Promastigotes were cultured in vitro at 26 °C in Schneider’s insect medium (Sigma Aldrich, St. Louis, MO, USA) supplemented with 20% heat-inactivated fetal calf serum (Eurobio, Les Ulis, France), and 0.1 μg/ml of 6-biopterin (Sigma Aldrich, St. Louis, MO, USA)**.** Every 4–6 days, the parasites were subcultured 1:10 in fresh medium and were kept in culture for a maximum of 20–25 passages.

Total DNA from parasite cells was isolated using the phenol-chloroform-isoamilic alcohol method [22]. RNA was isolated, using the Trizol reagent (Sigma-Aldrich, St. Louis, MO, USA), from 10 ml of *L. braziliensis* logarithmic phase promastigotes incubated further at 26 °C, 35 °C or 37 °C for 2 h.

For Western blot assays, logarithmic phase promastigotes, either grown at 26 °C or incubated further at 35 °C or 37 °C (either 2 or 4 h) were collected. After washing with cold phosphate-buffered saline (1× PBS) containing a protease inhibitor cocktail (Roche, Mannheim, Germany), cells were directly lysed in SDS-PAGE sample buffer.

### Cloning and sequence analysis of the *L. braziliensis* SCD6 and RBP42 genes

*LbSCD6* coding region was amplified from total DNA using the primers Lb2210F (5′-GAG CTC ATG CAC AAC GCC GTA GGC-3′) and Lb2210R (5′-CCC GGG TCA ACG CTG CTG GTA GCG T-3′), based on *LbrM.25.2210* gene sequence available at GeneDB database. These primers contain the *Sac*I and *Sma*I restriction sites (underlined in the sequence, respectively), which were introduced for cloning purposes. The resulting amplification fragment was 885 bp in length.

For amplification of *LbRBP42* coding region, the primers Lb3080F’ (5′-GGA TCC ATG GCT GCC GCT CAG CAA GTT-3′) and Lb3080Xho (5′-CTC GAG TTA CTC GCG GGC GCG CT-3′) were designed, based on *LbrM.30.3080* gene sequence available at GeneDB database. For cloning purposes, *Bam*HI or *Xho*I restriction sites (underlined in the sequence, respectively) were included. The resulting amplification fragment was 1017 bp in length. The PCR products were recovered from an agarose gel using Zymoclean Gel DNA Recovery Kit (Zymo Research, Irvine, CA, USA) and cloned into pCR2.1 plasmid (Invitrogen, Carlsbad, CA, USA), for the LbSCD6 gene, and pGEX-5×-3 plasmid (GE Healthcare, Chicago, USA), for the *LbRBP42* gene. The inserts of selected clones were sequenced using the Big Dye Terminators v3.1 kit (Applied Biosystem, Foster City, CA, USA) by automatic sequencing at the Servicio de Genómica (Parque Científico de Madrid, Universidad Autónoma de Madrid).

### Cloning and expression of the *L. braziliensis* SCD6 and RBP42 genes

The *LbSCD6* and *LbRBP42* coding regions were subcloned into the pQE30 expression plasmid (Qiagen, Hilden, Germany), and thermo-competent *E. coli* cells (M15 strain) were transformed with the corresponding ligation reaction. After colony selection, bacteria were grown overnight at 37 °C in 200 ml of LB medium supplemented with 100 μg/ml of ampicillin and 25 μg/ml of kanamycin. When the culture reached an A_600_ of 0.6, protein expression was induced by adding 1 mM isopropyl thio-β-galactopyranoside (IPTG); afterwards, bacteria were further incubated at 37 °C (2 h for rLbSCD6 expressing bacteria and 3 h for the rLbRBP42 expressing bacteria). The cells were harvested (15 min, 4696× *g* at 4 °C), and bacteria were lysed by sonication after suspending them in 10 ml of urea buffer (20 mM Tris-HCl, 0.5 M NaCl, 5 mM imidazole, 1 mM β-mercaptoethanol and 8 M urea; pH 8.0). The presence of the proteins in the supernatant was checked by SDS-PAGE.

Protein purification was carried out by affinity chromatography using a Ni^2+^-NTA-Agarose resin (QIAGEN, Hilden, Germany). After binding to the nickel-column, the proteins were refolded using a continuous decreasing gradient of urea buffer (from 8 M urea to no urea). Subsequently, rLbSCD6 protein was eluted in a buffer containing 20 mM Tris-HCl, 0.5 M NaCl, 1 mM β-mercaptoethanol and 300 mM imidazole (pH 8.0), whereas rLbRBP42 protein was eluted in the same buffer but containing only 200 mM imidazole. The concentrations of the refolded proteins were determined using the Micro BCA protein assay kit (Thermo Scientific, Waltham, MA, USA). Finally, the purification process and the purity of the proteins were checked by SDS-PAGE (see Additional file 1: Figure S1).

### Analysis of RNA binding capacity of SCD6 and RBP42 recombinant proteins by pull down assays

To determine the ability of rLbSCD6 and rLbRBP42 proteins to bind RNA, pull down assays were performed, following the protocol described by Nocua et al. [23] using as target the *L. braziliensis* 3′ *HSP70-II* UTR digoxigenin labeled region [10]. Briefly, increasing concentrations of rLbSCD6 and rLbRBP42 proteins (0.25, 1.25 and 6.25 pM) were incubated with 0.8 mg of His-tag isolation & Pull down dynabeads (Invitrogen, Carlsbad, CA, USA) in 1× binding buffer at room temperature (RT) for 15 min on continuous stirring. Unbound proteins were removed and free His-sites in the beads were blocked by adding 5 μg of recombinant *L. braziliensis* α-tubulin for 20 min. Then, the beads were incubated with 200 ng of digoxigenin-labeled *HSP70-II* 3′ UTR for 20 min in pull-down 1× buffer, and washed with pull down buffer 0.5×. A final wash, with DEPC-treated distilled water, was performed. Finally, the protein-nucleic acid complexes were eluted in pull down elution buffer by incubation at 90 °C for 5 min. These complexes were analyzed by dot immunoblotting and the signal was detected using the DIG luminescent detection kit (Roche, Mannheim, Germany).

### Prediction of RNA motifs in the 3′-UTR of the *L. braziliensis HSP70-II* gene

To identify potential *cis*-elements into the Lb*HSP70-II* 3′ UTR that might be involved in RNA-protein interactions, the MEME Suite web server [24] was used. This analysis was performed on the *HSP70-II* 3′ UTR of different *Leishmania* species (*Leishmania infantum*, *Leishmania mexicana*, *L. major* and *L. braziliensis*) to define conserved motifs*.* Additionally, a comparison with other *cis*-acting factors, previously characterized in either trypanosomatids or other species, was carried out. Finally, one motif was selected and chemically synthetized. In the synthetic oligoribonucleotide, named 3′ UTR-II.1 motif, a digoxigenin molecule was added at its 5′ end. The sequence of the 3′ UTR-II.1 motif is: 5′-AUGUCUUUUA UUUUUUUGUG UGUGUUUUAU AUUUUUCUCC UUUCGUACUA A-3′.

### Electrophoretic mobility shift assay (EMSA)

The interaction between rLbSCD6 or rLbRBP42 and the *L. braziliensis HSP70-II* 3′ UTR motifs were evaluated by EMSA assays. Briefly, different binding mixtures containing 12.5 nM digoxigenin labeled oligoribonucleotide (3′ UTR-II.1 motif) were used together with increasing concentrations (12.5, 25, 50 and 100 nM) of cold oligoribonucleotide. Afterwards, 3 pM (final concentration) of recombinant proteins was added, in a final volume of 20 μl using a binding reaction buffer (10 mM HEPES pH 7.3, 20 mM KCl, 6 mM MgCl_2_, 1 mM DTT, 0.05% NP-40, 1 μg/μl heparin and 40 U RNaseOUT (Invitrogen, Carlsbad, CA, USA)). The samples were incubated at RT for 20 min. Then, samples were loaded onto 6% native polyacrylamide minigels (Bio-Rad Laboratories, Hercules, CA, USA) in 0.5× TBE and run at 100 V for 1 h. Later, the gel was transferred onto a nylon membrane to proceed with the immunological detection of the complexes. Finally, the migration of the digoxigenin-labeled oligoribonucleotide was revealed by the DIG luminescent detection kit (Roche, Mannheim, Germany), following the protocol described by Nocua et al. [23].

Additionally, competition EMSA assays were performed using a cold irrelevant oligonucleotide, which corresponds to the antisense sequence of the 3′ UTR-II.1 motif. The sequence of the 3′ UTR-II.1 antisense motif is: 5′-UACAGAAAAU AAAAAAACAC ACACAAAAUA UAAAAAGAGG AAAGCAUGAU U-3′. In these assays, increasing concentrations (12.5, 25, 50 and 100 nM) of cold antisense oligonucleotide were added to the mixture containing 12.5 nM of digoxigenin labeled 3′ UTR-II.1 motif, and the recombinant proteins were added for a final concentration of 3 pM. Finally, the detection of complexes was performed as indicated above.

### Northern blot assays

Roughly 3.6 × 10^8^ promastigotes were used to isolate RNA by TRI reagent method (Sigma Aldrich, St. Louis, MO, USA) following manufacturer’s instructions. About 8 μg of total RNA were loaded onto 1.5% (*w*/*v*) low electroendosmosis agarose/MOPS/formaldehyde gels and, after electrophoretic separation, transferred onto nylon membranes. Probes used in these studies were prepared and labeled with digoxigenin during PCR amplification using specific primers for each gene and the PCR DIG Probe Synthesis kit (Roche, Mannheim, Germany). A probe of LbAmastin coding region was obtained based on the *LbrM.20.4320* gene, using the LbAmasFw (5′-ATG AAG CGG AGT GTT CCC ATT C-3′) and LbAmasRv (5′-CTA CTC CTC CTC CTC CTT TTG TGT G-3′) primers. Hybridization and immunological detection were performed using the Detection Starter kit II (Roche, Mannheim, Germany) according to the manufacturer’s instructions. Finally, membranes were exposed on X-Ray film (AGFA, Mortsel, Belgium).

### Western blot assays

A total of 1 × 10^8^ cells were harvested by centrifugation and the pellet washed with PBS containing a protease inhibitor cocktail (Roche, Mannheim, Germany). Cells were then lysed in loading buffer (62.5 mM Tris-HCl pH 6.8, 7.5 mM EDTA (pH 8.0), 2% sodium dodecylsulfate (SDS), 10% glycerol, 5% β-mercaptoethanol, and 0.1% bromophenol blue) and briefly sonicated. Per lane, samples derived from 5 × 10^6^ parasites were loaded. After protein separation on 10% SDS-PAGE gels and transferring onto nitrocellulose membranes, the blots were blocked by incubation for 1 h in blocking solution (5% skimmed milk dissolved in a solution of PBS supplemented with 0.05% Tween 20 (PBS-T)). The membranes were incubated with rabbit polyclonal anti-LbSCD6 or anti-LbRBP42 antibodies diluted at 1:10,000 in blocking solution. Binding of the primary antibodies was detected by incubating with horseradish peroxidase-conjugated anti-rabbit igG antibodies diluted at 1:5000; after washing of the membranes, they were developed with Immun-Start HRP Kit (Bio-Rad Laboratories, Hercules, CA, USA). Finally, X-Ray film (AGFA, Mortsel, Belgium) were exposed on the membranes. The autoradiographs were quantified using the Amersham Imager 600 program together with the ImageQuant TL software (GE Healthcare, Chicago, USA). The densitometric values were normalized using autoradiographs obtained after incubation of the same blots with an anti-α-tubulin antibody (1:8000).

All polyclonal antibodies were produced in New Zealand white rabbits at the Centro de Investigación y Desarrollo (Barcelona, Spain) using the corresponding purified recombinant proteins as immunogens.

### Subcellular fractionation procedure

Subcellular fractionation protocol was adapted from the method described elsewhere [25]. Briefly, 10^7^ *L. braziliensis* promastigotes were incubated at either 26 °C or 37 °C for 2 h. Cells were then harvested by centrifugation and extensively washed with cold PBS. Cells were lysed in 1.5 ml of pre-chilled B1 buffer (100 mM Tris-HCl pH 7.4, 20 mM KCl, 12.5 mM MgCl_2_) supplemented with 1 mM PMSF, protease inhibitor cocktail (Roche, Mannheim, Germany), 5 mM β-mercaptoethanol, and 10% NP-40. Parasites were carefully resuspended by pipetting up-down 10 times and centrifuged at 5000× *g* for 2 min at 4 °C. The cytoplasmic fraction (supernatant) was collected and again centrifuged twice under the same conditions. Nuclear enriched fraction (pellet) was centrifuged again at 7000 *g* for 2 min at 4 °C. The fractions were separated on 12% SDS-PAGE gels and the protein localization was detected by Western blot using ​appropriate antibody (diluted 1:5000 for both proteins). As control of the fractionation process, the fractions were incubated with anti-HSP70 (cytoplasmic protein) and anti-Histone 3 (nuclear protein) antibodies diluted at 1:5000 and 1:2500, respectively.

### Confocal microscopy

Parasites were washed three times with precooled PBS. Next, they were resuspended at a density of 5 × 10^6^ parasites/ml in PBS. The cells were fixed with 3.7% paraformaldehyde solution for 20 min at RT followed by immersion in precooled methanol at 20 °C for 5 min. Once fixed, the cells were rehydrated with two changes of PBS. Next, they were treated with 1 mg/mL RNase A (Sigma Aldrich, St. Louis, MO, USA) and incubated for 30 min at 37 °C in a wet chamber. Later, the samples were washed three times with PBS, blocked by incubation during 5 min with TB buffer (PBS containing 0.1% Triton X100 and 0.1% BSA) and incubated for 1 h with the polyclonal anti-LbSCD6 (1:500) or anti-RBP42 (1:250) antibodies at RT. Subsequently, three washes were performed and the samples were incubated with Alexa Fluor 488 donkey anti-rabbit IgG (Invitrogen, Carlsbad, CA, USA) at a 1:1000 dilution for 1 h at RT in the dark. The nuclear DNA was stained by incubation with 2.5 μg/ml propidium iodide for 30 min at RT. After three washes, the samples were dried and mounted using ProLong antifade reagent (Invitrogen, Carlsbad, CA, USA). The samples were examined using an Olympus FluoView™ FV1000 confocal microscope (Olympus, Center Valley, PA, USA).

## Results

### Structural analysis and modeling of LbSCD6 indicated that it belongs to the Lsm family of RNA-binding proteins

**Fig. 1:**
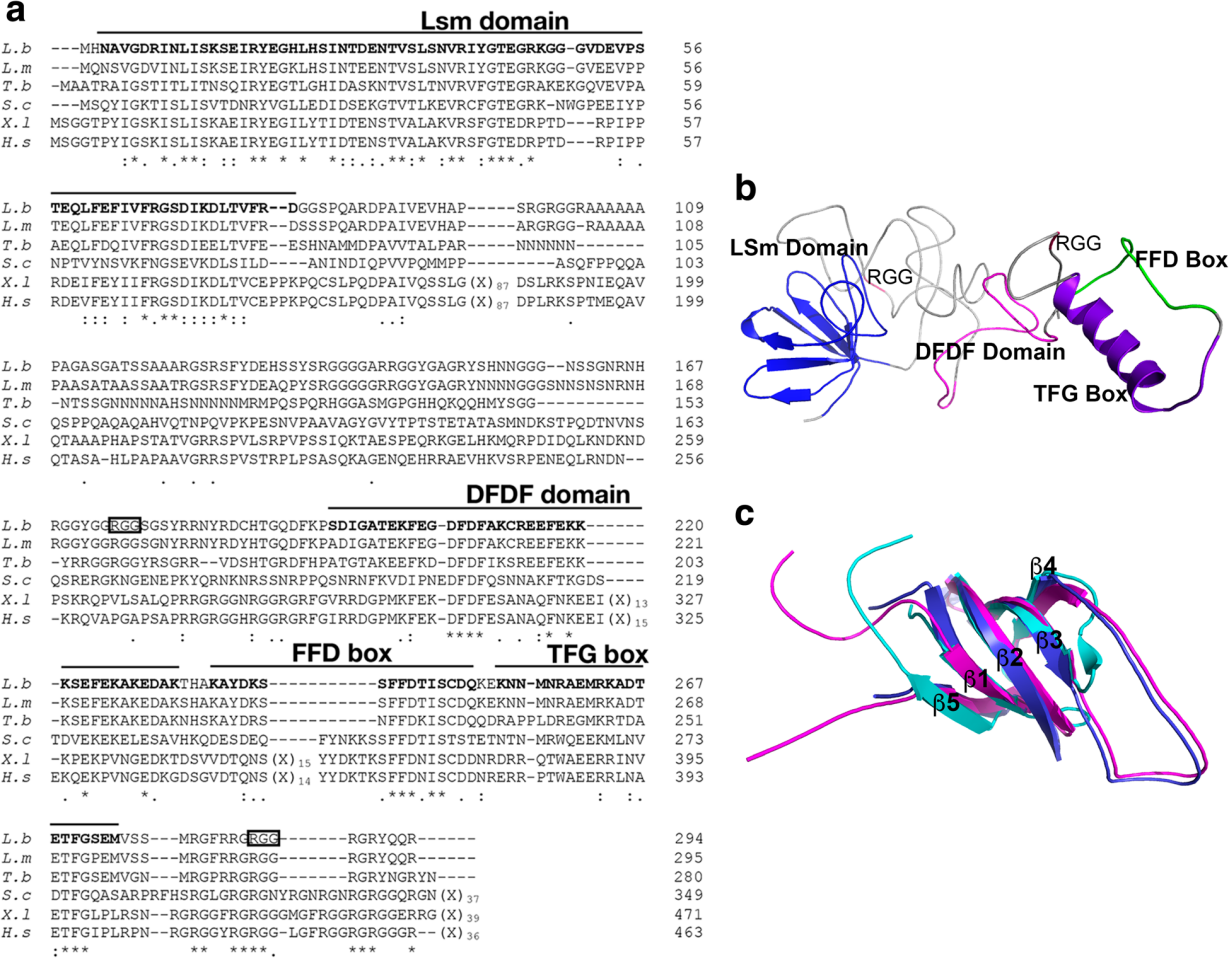
Structure of *L. braziliensis* SCD6 protein and molecular modeling. **a** Multiple sequence alignment of SCD6 protein from trypanosomatids and evolutionarily distant organisms. Protein codes: XP_001565722 (*L. braziliensis*), CAJ04780 (*L. major*), XP_828147.1 (*T. brucei*), P45978 (*S. cerevisiae*), BAF36055 (*X. laevis*) and NP_056393.2 (*H. sapiens*). **b** Tridimensional structure of *L. braziliensis* SCD6 protein generated by Phyre2. The different domains and boxes present in this protein are indicated in color as follows: Lsm domain in blue, DFDF domain in magenta, the RGG regions in red, FFD Box in green and TFG Box in purple-blue. **c** An overlay of the structures of the LbSCD6-LSm domain (*blue*) with the LSm domain of *D. melanogaster* TraI (*magenta*, PDB: 2VXE) and LSm domain of *H. sapiens* Edc3 (*cyan*, PDB: 2VC8)

Preliminary BLAST searches showed a certain degree of sequence identity in its primary sequence not only with orthologues protein in other *Leishmania* species and related trypanosomatids, but also with evolutionarily divergent organisms like *Saccharomyces cerevisiae*, *Xenopus laevis* and *Homo sapiens* (Fig. 1a). The percentages of sequence conservation agreed with the evolutionary distance separating the species analyzed. Thus, the LbSCD6 primary amino acids sequence showed 89.2% of sequence identity (96.6% of sequence similarity) with the *Leishmania major* orthologue, 48.8% of sequence identity (73.7% of similarity) with the *T. brucei* orthologue, but only 27–28% of sequence identity (45.7–56.6% of similarity) with the putative orthologues in *Xenopus*, yeasts and humans (Additional file 2: Table S1).

A search of protein domain was done using the InterProScan tool. Two distinguishable domains, which are typically found in proteins of the Sm or Like-Sm family proteins, were found. The N-terminal 79 amino acids of LbSCD6 conform a Like-Sm domain (InterPro domain IPR010920, Lsm domain). The second identified motif is in the middle of the molecule and corresponds to the DFDF domain (InterPro domain IPR025762). Furthermore, at the C-terminal region, two boxes, FFD (InterPro domain IPR025761) and TFG (InterPro domain IPR025768), were identified (Fig. 1a).

To gain additional structural insights, a molecular modeling was done using the Phyre2 server (Fig. 1b), and validated by the Ramachandran plot (Additional file 3: Figure S2). Interestingly, the LbSCD6-Lsm domain (Fig. 1b) adopted a five-stranded β-barrel-like structure, which is a distinctive feature of the Sm domains, but it lacks the characteristic α helix structure found at the N-terminus in other Sm domains [26]. Furthermore, a structural-based alignment of the LbSCD6-LSm region with the LSm domains of human EDC3 and *D. melanogaster* TraI proteins (Fig. 1c) revealed that LbSCD6-Lsm domain fits perfectly in the conserved segments Sm1 (composed by the 1β to 3β sheets) and Sm2 (4β and 5β), defined in this family of proteins [27]. On the other hand, the DFDF domain, defined by the DFDF-x(6)-F motif, has a predicted helical structure enriched in polar and charged residues [26]. In LbSCD6, this motif is well conserved (DFDFAKCREEF) and, as occurs in the RAP55 protein family of vertebrates and the *Drosophila melanogaster* TraI protein [27, 28], contains two RGG repeats flanking the DFDF domain and FFD-TGF boxes (Fig. 1). It has been described that these RGG regions enhance protein-protein interactions [28]. Finally, the boxes FFD (Y-x-K-x(3)-FFD-x-[IL]-S) and TFG ([RKH]-x(2–5)-E-x(0–2)-[RK]-x(3–4)-[DE]-TFG [29] are also well conserved in LbSCD6 (Fig. 1a, b). Moreover, the TFG box in LbSCD6 adopts an α-helix conformation as described for other proteins of LSm family that present this box [29].

### Structural features and modeling of LbRBP42 allowed its adscription to the NTF2 family of RNA-binding proteins

**Fig. 2:**
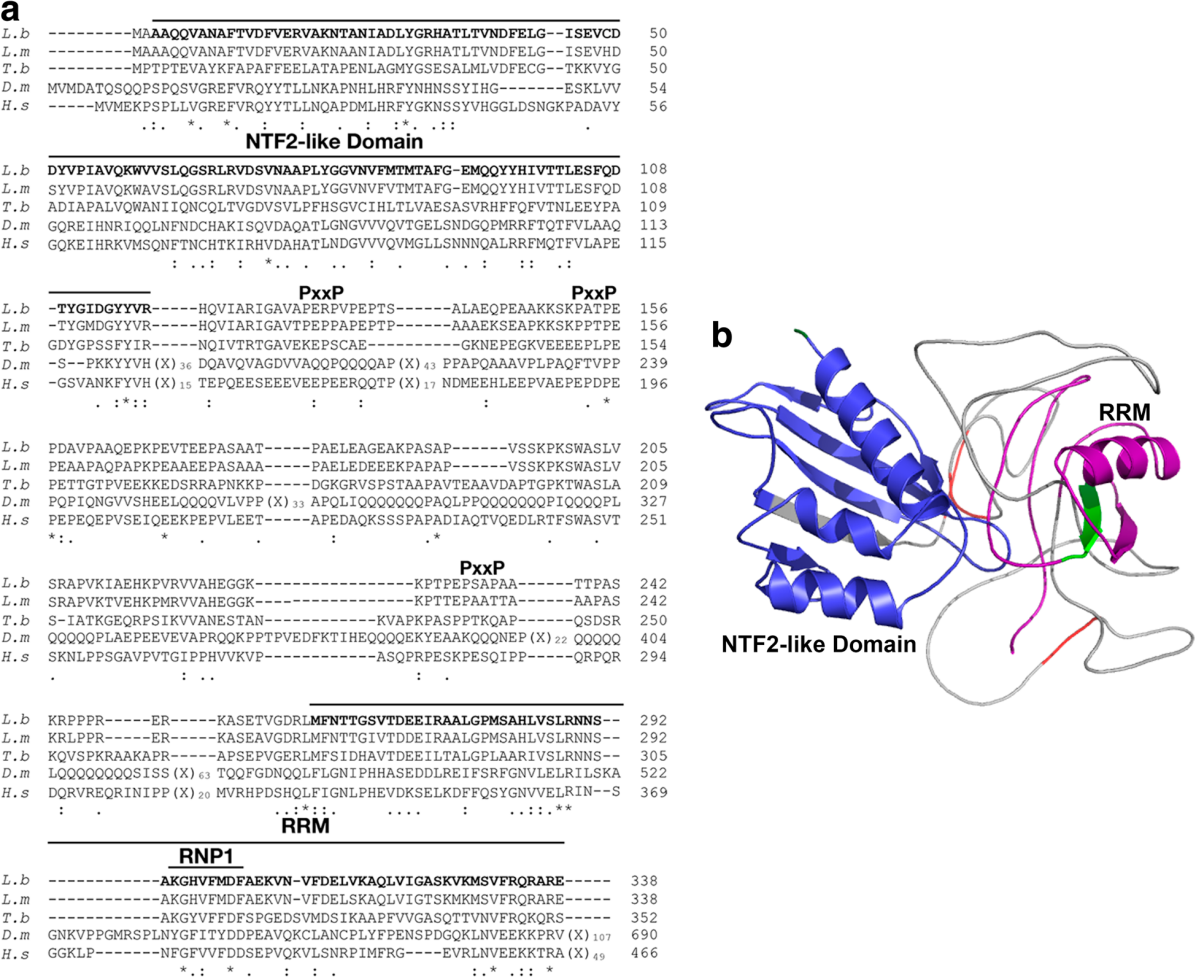
Structure of *L. braziliensis* RBP42 protein and molecular modeling. **a** Multiple sequence alignment of RBP42 orthologues from trypanosomatids and evolutionarily distant organisms. Protein codes are: XP_001566933.1 (*L. braziliensis*), XP_001684917 (*L. major*), XP_845561.1 (*T. brucei*), AF231031_1 (*D. melanogaster*) and Q13283 (*H. sapiens*). **b** Tridimensional structure of *L. braziliensis* RBP42. The different domains present in this protein are shown using the following colors: NTF2-like domain in *blue*, PxxP motifs in *red* and RNA recognition motif (RRM) in *purple*. Within this domain, the signature sequence RNP1 is shown in *green*

BLAST searches indicated that LbRBP42 is conserved in other *Leishmania* species and in *T. brucei*, but also in organisms evolutionarily more distant as *D. melanogaster* and *H. sapiens* (Fig. 2; Additional file 2: Table S1). A search for structural domains revealed that the N-terminal 114 amino acids conform the NTF2-like protein domain (IPR032710). In addition, an RRM motif was predicted in the C-terminal end 73 amino acids of LbRBP42 (Fig. 2).

Molecular modeling of the LbRBP42 showed that the NTF2-like domain conforms the typical structure of this domain (Fig. 2b), which is composed of three α helices lined up against a β sheet made up by five β strands [30]. Regarding the RRM domain, in the mammalian orthologue to LbRBP42, G3BP, the domain contains two conserved helical regions [30]. However, in the LbRPB42, as it was also noticed for TbRPB42 [18], the RMM domain would be truncated and presents only one helix. In fact, the RRM-like domain of LbRBP42 seems to have only the RNP1 signature, whose sequence corresponds to KGHVFMDF (Fig. 2b), lacking the other signature, RNP2, which usually is also found in the RRM domain. On the other hand, three PxxP motifs (Fig. 2) were found in LbRBP42 protein; the presence of this motif, repeated several times, is also a hallmark of the members of the G3BP protein family [31].

### *Leishmania braziliensis* LbSCD6 and LbRBP42 recombinant proteins interact in vitro with RNA molecules

To analyze whether LbSCD6 and LbRBP42 proteins can directly bind RNA molecules, we conducted pull-down assays using the corresponding recombinant proteins expressed in *E. coli*. For both proteins, high levels of expression were observed after induction with IPTG (Additional file 1: Figure S1); unfortunately, these proteins were mainly present in the insoluble fraction (i.e. inclusion bodies). Therefore, it was necessary to solubilize them by using urea-containing buffers, and refolding after their purification by affinity chromatography in nickel-column.

**Fig. 3:**
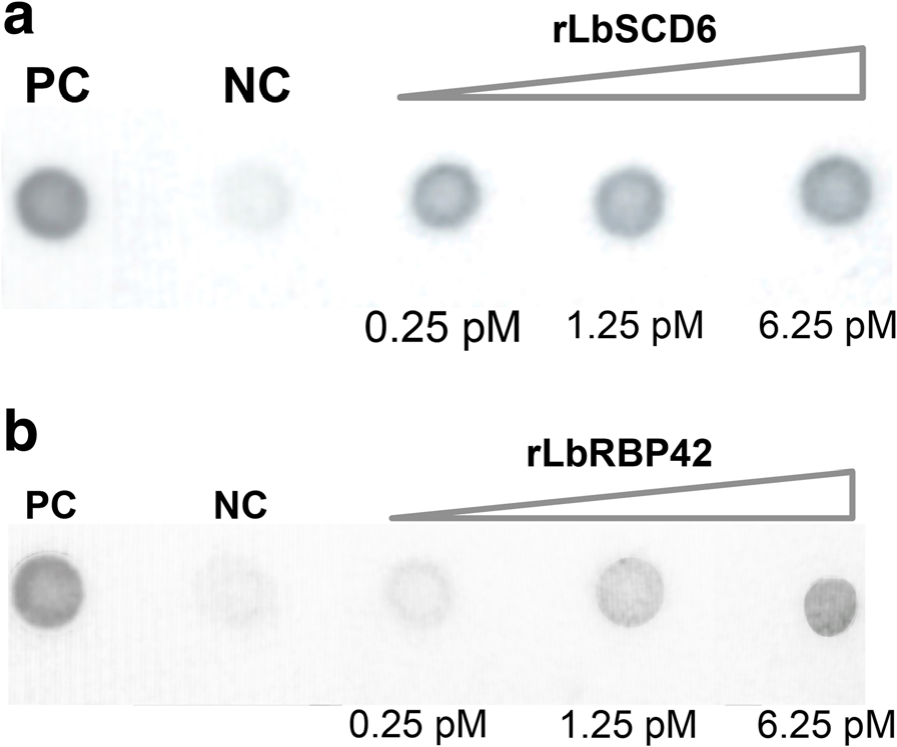
Analysis of RNA-binding capacity by pull down assays. **a** Analysis of rLbSCD6. **b** Analysis of rLbRBP42. Two hundred nanograms of digoxigenin labeled HSP70-II 3′ UTR were incubated with beads containing different amounts of protein. After washing out of the unbound RNA, eluted complexes were deposited on the membrane and the bound RNA was monitored by an antidigoxigenin antibody. RPA1 protein (6.25 pM) was used as a positive control (PC) [23], and Lbα-tubulin as a negative control (NC)

As the *L. braziliensis* SCD6 and RBP42 endogenous proteins were identified by their interaction with the 3′ UTR of *HSP70-II* transcript in the initial screening assay [10], we prepared a digoxigenin-labeled RNA probe expanding the complete HSP70-II 3′ UTR. Interestingly, these assays showed that both rLbSCD6 (Fig. 3a) and rLbRBP42 (Fig. 3b) proteins are able to directly interact with RNA molecules. As a positive control the rLbRPA1 protein was used, whose RNA binding capacity was recently demonstrated [23]. Additionally, to exclude non-specific interactions, beads coated with Lbα-tubulin were used as negative control (Fig. 3a, b, dot NC). In agreement with the fact that α-tubulin protein does not have RNA binding activity, no interaction with the labeled RNA was observed in these samples. Overall, these results suggested that the recombinant proteins were refolded correctly during the in vitro purification process and, more importantly, that LbSCD6 and LbRBP42 proteins, according to the structural predictions, would be RNA-binding molecules.

### rLbSCD6 and rLbRBP42 specifically interact with a defined motif into the 3′ UTR of the *HSP70-II* transcript

**Fig. 4:**
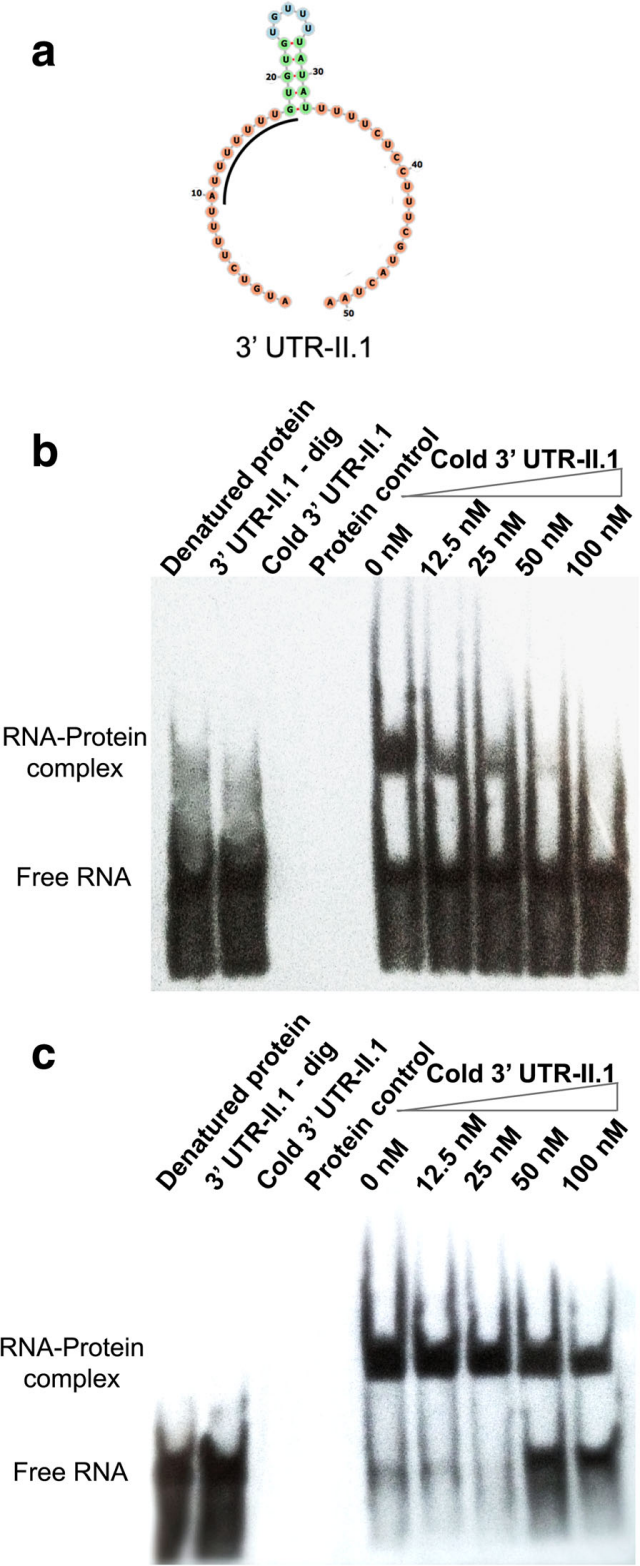
Analysis of the rLbSCD6 and rLbRBP42 binding capacity to the 3′ UTR-II.1 motif. **a** RNA secondary structure of the 3′ UTR-II.1 motif; the putative binding sequence is underlined. The structure motif was obtained by RNAfold. EMSA assays were done using a stable concentration of rLbrSCD6 (**b**) and rLbRBP42 (**c**) proteins. Different mixtures of both labeled and cold 3′ UTR-II.1 motif were used. These mixtures contained a constant concentration of the labeled ribonucleotide and different amounts (1-, 2-, 4- or 8-fold excess) of the non-labeled (cold) ribonucleotide (Lanes 6–9). In lane 5, the reaction was done only in the presence of the 3′ UTR-II.1-dig ribonucleotide. As control of unspecific binding, the denatured recombinant proteins were used

After demonstrating the direct interaction of both proteins with RNA molecules, further experiments were undertaken to find candidate *cis*-acting sequences involved in this interaction. For this purpose, a prediction of secondary structure in the HSP70-II 3′ UTR was carried out. The existence of a long loop called our attention, and we decided to analyze whether this motif, named 3′ UTR-II.1, might be a possible recognition site for these *L. braziliensis* RNA-binding proteins. This motif consists of an adenylate/uridylate-rich element (ARE), located at positions 252–302 of the *HSP70-II* 3′ UTR [22]. Hence, we decided to synthetize an oligoribonucleotide expanding this sequence to directly analyze the interaction of this motif with LbSCD6 or LbRBP42. Secondary structure predictions indicated that this motif conforms a single strand loop containing a small stem in the middle of the structure (Fig. 4a). This structure is maintained when predictions are made either using the complete *HSP70-II* 3′ UTR or solely the 3′ UTR-II.1 sequence.

Electrophoretic mobility shift assays (EMSA), using the digoxigenin-labeled oligoribonucleotide 3′ UTR-II.1 motif in the presence of the recombinant proteins, were used to analyze the possible interaction between the *L. braziliensis* SCD6 (Fig. 4b) and RBP42 (Fig. 4c) proteins with the selected motif. Retardation bands were observed in both assays as a demonstration of an interaction of the proteins with the 3′ UTR-II.1 motif. Mobility shift was not observed when the denatured proteins were used as negative control. In addition, competition assays were carried out using increasing amounts of non-labeled 3′ UTR-II.1 oligoribonucleotide (Fig. 4b, c). Interestingly, although both proteins interacted with the 3′ UTR-II.1 motif, the competition assays suggested that LbRBP42–3′ UTR-II.1 interaction is more stable than that established with the LbSCD6 protein, as the complex formed between rLbSCD6–3′ and UTR-II.1-dig was completely competed by the presence of a 1-fold excess of cold 3′ UTR-II.1 motif (Fig. 4b). In contrast, for rLbRBP42 protein, a decrease in the amount of rLbRBP42–3′ UTR-II.1-dig complex was only observed when the unlabeled 3′ UTR-II.1 motif was 4-fold in excess (Fig. 4c).

**Fig. 5:**
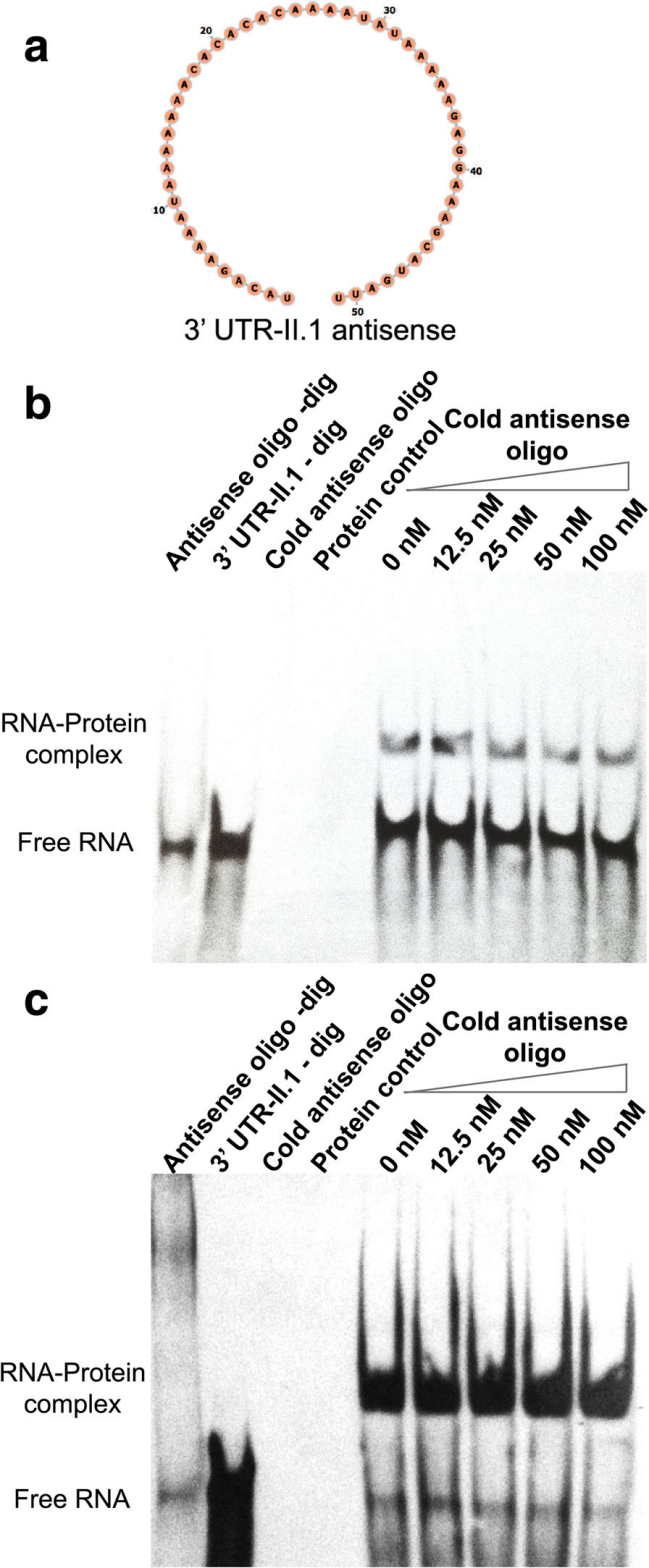
Analysis of rLbSCD6 and rLbRBP42 binding capacity to the 3′ UTR-II.1-antisense motif. **a** Secondary structure of the 3′ UTRII.1-antisense motif. Competitive EMSA assays were done using a constant concentration of the rLbSCD6 (**b**) and rLbRBP42 (**c**) proteins and a mixture with both 3′ UTR-II.1-dig and cold antisense ribonucleotides. These mixtures contained a constant concentration of 3′ UTR-II.1-dig ribonucleotide and different amounts (1-, 2-, 4- or 8-fold excess) of the antisense ribonucleotide. In lane labeled ‘0 nM’, the reaction mixture contained the 3′ UTR-II.1-dig ribonucleotide, but the antisense ribonucleotide was not present. To confirm the non-interaction between proteins and the antisense motif, an interaction assay was performance using the digoxigenin-labeled antisense ribonucleotide (lane antisense oligo-dig)

To gain further insights into the sequence specificity in the recognition of the 3′ UTR-II.1 motif by rLbSCD6 and rLbRBP42, we designed an oligoribonucleotide competitor, named 3′ UTR-II.1 antisense, which consists of the reverse sequence regarding that of the 3′ UTR-II.1 motif. Structural predictions indicated that the 3′ UTR-II.1 antisense sequence does not adopt any secondary structure (Fig. 5a). As shown in Fig. 5, the binding of either rLbSCD6 (Fig. 5b) or rLbRBP42 (Fig. 5c) to the 3′ UTR-II.1 motif was not inhibited by an excess of the 3′ UTR-II.1 antisense oligoribonucleotide. Therefore, these results suggested that the interaction between the recombinant proteins and 3′ UTR-II.1 motif would be mainly sequence-specific, rather than dictated by a structural recognition of single-stranded RNAs.

### mRNA levels for *L. braziliensis* SCD6 and RBP42 are affected by changes in parasite growth temperature

**Fig. 6:**
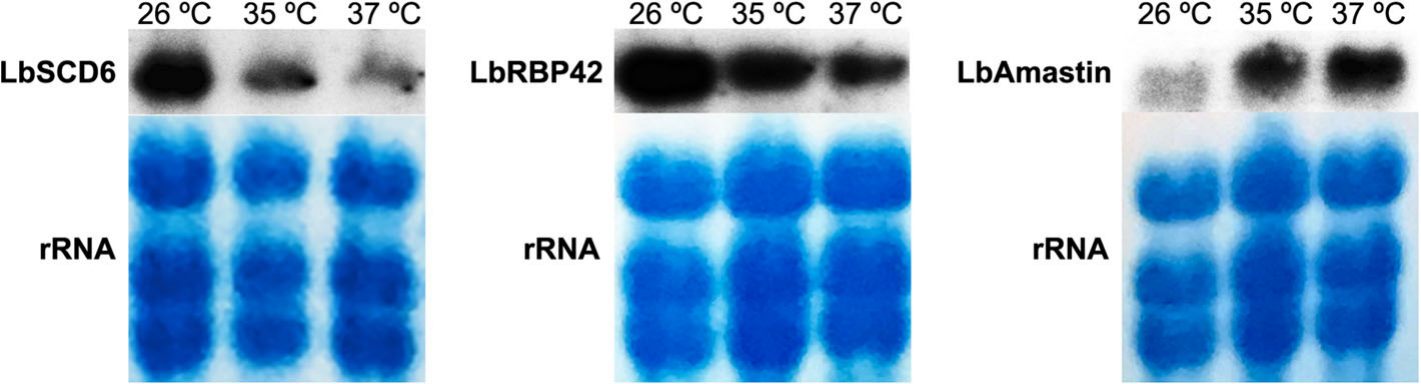
Analysis of LbSCD6 and LbRBP42 transcript levels in promastigotes growing at normal or heat-shock conditions. Total mRNA was obtained from promastigotes incubated for 2 h at the indicated temperatures. A probe derived from the amastin gene *LbrM.20.4320* was used as a heat-shock inducible gene. Equal loading was verified by staining the RNA blots with methylene blue prior to Northern blot hybridizations (rRNA panels)

It is well known that temperature shifts cause differential gene expression and stage transformation in *Leishmania* [32]. Hence, we evaluated the effect of either moderate (35 °C) or severe (37 °C) heat-shock treatments on the steady-state levels of the mRNAs coding for *L. braziliensis* SCD6 and RBP42 proteins. Thus, promastigotes growing at the mid-logarithmic phase were further incubated for 2 h at either 26 °C, 35 °C or 37 °C. Afterwards, RNA was extracted and assayed by Northern blot using probes derived from *L. braziliensis SCD6* and *RBP42* genes (Fig. 6). As a control, the RNA levels derived from the *L. braziliensis* amastin gene *LbrM.20.4320* were analyzed. It is well-known that the expression levels of *Leishmania* amastin genes are higher in amastigotes than in promastigotes and that their levels accumulate after incubation of the parasites at heat-shock temperatures [33]. As expected, it was observed an increased expression of amastin mRNAs in the *L. braziliensis* parasites incubated at elevated temperatures (Fig. 6). In contrast, the steady-state mRNA levels for LbSCD6 and LbRBP42 showed a marked decrease after parasite incubation for 2 h at heat-shock temperatures, being more pronounced at 37 °C. These results showed a downregulation of SCD6 and RBP42 mRNAs levels during the adaption process of *L. braziliensis* to the mammalian host temperature.

### LbSCD6 and LbRBP42 steady-state protein levels remain unaffected by changes in the temperature of growth

**Fig. 7:**
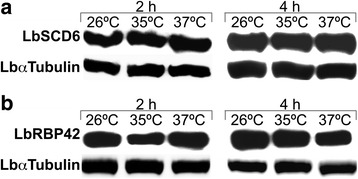
LbSCD6 and LbRBP42 protein levels in promastigotes growing at normal or heat-shock conditions. Analysis of LbSCD6 (**a**) and LbRBP42 (**b**) protein expression. Total protein extracts were obtained from promastigotes incubated for 2 or 4 h at the indicated temperatures. The relative protein levels were calculated after normalization with the amount of *L. braziliensis* α-tubulin protein present in each lane

Given the temperature-induced decrease in the levels of transcripts coding for LbSCD6 and LbRBP42, we assessed the relative abundance of each protein in parasites incubated at 26 °C, 35 °C and 37 °C for either 2 or 4 h (Fig. 7). In these assays, the immunological detection of α-tubulin was used as load control. No significant changes in the protein levels of LbSCD6 (Fig. 7a) and LbRBP42 (Fig. 7b) were observed in the parasites incubated at heat-shock conditions regarding their levels at 26 °C. Further experiments, analyzing the de novo protein synthesis and/or the protein turnover, should be done to conciliate the dramatic temperature-induced decrease in the mRNA levels with the finding that steady-state levels of LbSCD6 and LbRBP42 proteins remain unaffected after incubating the parasites at heat-shock temperatures.

### LbSCD6 and LbRBP42 are cytoplasmic proteins and migrate to nuclear periphery under heat shock

**Fig. 8:**
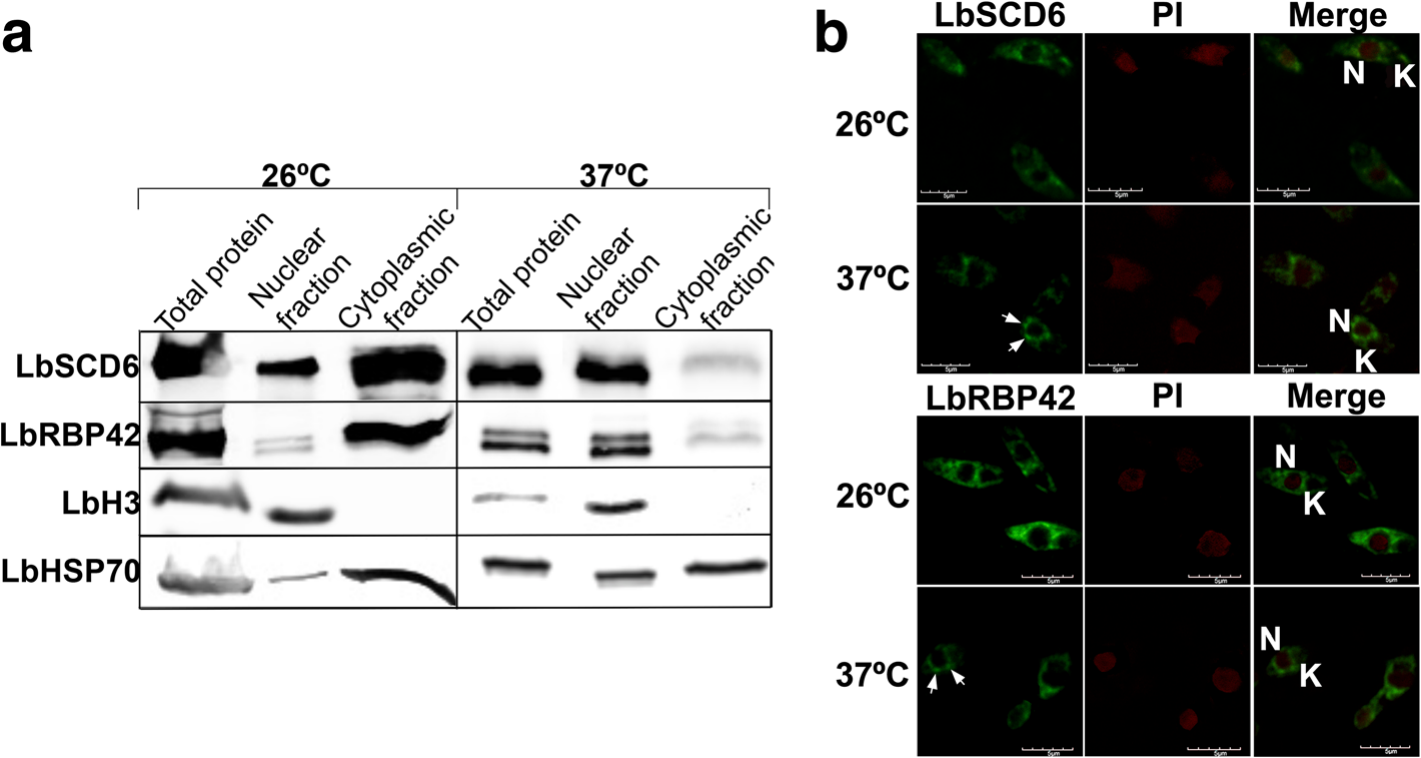
Analysis of the cellular localization of LbSCD6 and LbRBP42 proteins in normal and heat-shock conditions. **a** Western blot analyses of nuclear and cytoplasmic fractions obtained from parasites incubated 2 h at 26 °C or 37 °C. To monitor the fractionation process, fractions were also analyzed by the presence of Histone 3 (nuclear marker) and HSP70 (cytoplasmic marker). **b** Confocal microscopy using either anti-LbSCD6 or anti-LbRBP42 antibodies in parasites incubated at 26 °C or 37 °C. Arrows point to the protein loci observed around the nucleus in parasites subjected to heat shock. Nuclei were visualized by propidium iodide (PI) staining. *Abbreviations*: N: nucleus, K: kinetoplast. *Scale-bars*: 5 μm

Orthologous to SCD6 and RBP42 proteins have been found to be in the cytoplasm of different organisms, being associated with RNP granules such as stress granules and P-bodies [18, 28, 34]. However, as no previous data exist regarding the subcellular location of these proteins in *Leishmania* parasites, we first analyzed the LbSCD6 and LbRBP42 location by biochemical subcellular fractionation at either normal growth conditions or after a heat-shock treatment at 37 °C for 2 h (Fig. 8a). As controls of the fractionation process, the locations of HSP70 (a cytoplasmic protein) and histone H3 (a nuclear protein) were also determined in the different fractions. Remarkably, a relocation of LbSCD6 and LbRBP42 from the cytoplasm to the nuclear fraction was observed after heat-shock treatment of the parasites. Thus, while both proteins are located essentially in the cytoplasm at 26 °C, with a small fraction of LbSCD6 associated with the nuclear fraction, the proteins seem to translocate to the nucleus after incubation of the parasites for 2 h at 37 °C. A fraction of HSP70 was found in the nuclear fraction after the heat-shock treatment; this movement from the cytoplasm to the nucleus of HSP70 has been described in other organisms [35]. To confirm the observed relocation of LbSCD6 and LbRBP42 proteins when the parasites are submitted to heat shock, a confocal microscopy analysis was carried out (Fig. 8b). It was found that whereas at 26 °C, LbSCD6 and LbRBP42 are evenly distributed in the cytoplasm, after the heat-shock treatment, both proteins concentrate around the nucleus (see arrows in Fig. 8b). Nonetheless, colocalization between these proteins and the nuclear DNA was not observed, suggesting that these proteins may be located at the nuclear periphery.

## Discussion

The study of RNA-binding proteins is of great importance given the role played by these *trans*-acting factors in the global context of gene expression. In this regard, RNA-binding proteins in trypanosomatids, in which gene transcription is not regulated, have to be central players in the mechanisms that regulate gene expression by their interaction with the untranslated regions in mRNAs [7, 9].

In this work, we carried out the molecular characterization of LbSCD6 and LbRBP42 proteins, which were identified in a previous work of our group because their association at 26 °C with the 3′ UTR of *L. braziliensis HSP70-II* mRNAs [10]. For both proteins, structural motifs related to RNA-binding activity were found. Thus, LbSCD6 contains an Lsm domain, a common feature of the members of the large Sm/Lsm protein family, mostly involved in RNA metabolism. Also, it has been reported that the Lsm fold containing-proteins act as chaperones, facilitating a variety of protein-protein interactions, which allows this family of proteins to assemble into different protein complexes that interact with RNA [27]. Recently, in *T. brucei*, the homologue to LbSCD6 has been described as one of the main components of P-bodies and stress granules, and it has been demonstrated that the Lsm domain is both necessary and sufficient for the formation of these granules [36]. Additionally, the LbSCD6 protein possesses a DFDF domain (Fig. 1), rich in polar and charged residues that could be involved in the direct interaction with RNA and/or highly charged polypeptides, which are commonly found in the ribonucleoprotein complexes [26]. More often, proteins that contain a DFDF domain always have an N-terminal Lsm domain [26, 29]; this finding suggests an evolutionarily conserved functional link between both domains [28]. Finally, several RGG repeats were also observed in the LbSCD6 structure (Fig. 1). These repeats, usually found at the C-terminus of proteins, mediate protein-RNA and/or protein-protein interactions, contributing to the assembly of RNP complexes. These interactions can be driven by the positive charge of the arginine amino acid presents in RGG repeats [37]. In yeast, these repeats are involved in the modulation of SCD6 protein translation, as its RGG motif interacts with eIF4G, preventing the formation of the 48S pre-initiation complex [37]. On the other hand, it has been described that this motif is required for the formation of many granules in *T. brucei* [36].

Regarding the structural motifs found in LbRBP42 protein, it presents the RRM domain, a common RNA binding domain found in eukaryotic RNA-binding proteins. This domain is found in splicing factors, poly-A-binding proteins (PABP) and the cap-binding proteins, among others [38]. In kinetoplastids, about 75 proteins with RRM domain have been described, of which only a few present clear homologues in other eukaryotes [39]. The RRM domain in LbRBP42 possesses a conserved signature, RNP1, consisting of eight residues, mainly aromatic and charged amino acids. It has been described that the side chains of these amino acids interact directly and specifically with the RNA [40]. Likewise, it has described that RRM domains can also mediate protein-protein interactions [40]. Another domain, found in the N-terminal region moiety of LbRBP42, was a NTF-2 like domain (Fig. 2). This domain is named because of its structural homology with the NTF2 protein, which is a small homodimeric protein involved in nuclear-cytoplasmic transport through the nuclear pore complex [41]. Considering the presence of this domain in LbRBP42, a role in the *Leishmania* nuclear transport for this protein may be postulated. On the other hand, the RRM and the NTF-2 like domains present also in the mammalian G3BP orthologue have been reported as responsible for the recruitment of this protein into the stress granules [42]. Finally, proline rich motifs (PxxP), also found in the LbRBP42 structure (Fig. 2), represent the minimal consensus sequence of the so-called proline rich region, which has been implicated in protein-protein interactions [43].

Structural analyses indicated that both proteins would be RNA binding proteins; thus, experimental approaches were designed to address the capacity of LbSCD6 and LbRBP42 proteins to directly interact with RNA molecules. First, pull down assays using the *HSP70-II* 3′ UTR as bait were carried out, and the results confirmed the interaction of both proteins with this RNA molecule. Secondly, looking for *cis*-acting sequences, with which these proteins could be interacting, a search for thermodynamically stable motifs in the HSP70 3′ UTR-II region was undertaken. A stable loop containing an AU-rich element (ARE) was selected. ARE sequences are often involved in determining mRNA stability in eukaryotes, affecting transcripts translation either positively or negatively [44]. Moreover, different studies have showed that both RRM and Lsm domains mainly interact with ARE elements [45]. In agreement, in this work, we have demonstrated that LbSCD6 and LbRBP42 stably interact with the ARE element present in the 3′ UTR of HSP70-type II mRNAs. Remarkably, this 3′ UTR-II.1 motif contains the AU_7_G sequence (Fig. 4a), which fits the previously reported Lsm interaction consensus site RAU_n_GR (R stands for purine nucleotide) [46].

Taking into account that these proteins were identified for their interaction with the HSP70-II 3′ UTR at 26 °C by pull down assays [17] and considering that HSP70-II transcripts are only translated when the parasites are submitted to heat-shock treatment (37 °C) [47], we looked for insights on a possible role of these proteins in the regulation of *HSP70-II* expression. Thus, searching for clues, we first analyzed whether the expression of LbSCD6 and LbRBP42 would be modulated by heat shock as HSP70 genes do. Interestingly, the steady-state levels of both transcripts were significantly reduced after incubation of *L. braziliensis* promastigotes at heat-shock temperatures. However, the levels of LbSCD6 and LbRBP42 proteins remained unaffected along the heat-shock treatment. These results suggest that these proteins may have long half-lives, but further experiments should be done to analyze a transient repression of de novo synthesis of these proteins during heat shock. Additionally, the regulation of these proteins should be studied in parasites adapted for transmission in the fly and survival in the mammalian host to exclude the possibility that a relaxation in the regulatory mechanisms had occurred during the axenic passage of the *L. braziliensis* strain used in this work.

Additionally, considering the functions assigned to this class of proteins in different organisms regarding the formation and transit of cytoplasmic RNP granules that have been proposed for this class of proteins in different organisms [16, 18, 48], we explored the subcellular location of LbSCD6 and LbRBP42 under both physiological and heat-shock conditions. Our results indicated that both proteins are scattered mainly in the cytoplasm during normal growth, but, under heat-shock conditions, the proteins formed aggregates that accumulate around the nucleus. A similar behavior was reported for the SCD6 homologue in human cells, the protein is diffusely observed throughout the cytoplasm, but when the cells are submitted to oxidative stress, the proteins are localized in discrete cytoplasmic foci (stress granules and P bodies) [14]. In addition, overexpression of SCD6 in *T. brucei* generates granules, which are not equally distributed throughout the cytoplasm but appeared enriched in regions adjacent to the nucleus [36]. Regarding RBP42, the *T. brucei* orthologue has been found by immunofluorescence microscopy in some foci at the nuclear periphery [18].

## Conclusions

The molecular and biochemical characterization of *L. braziliensis* SCD6 and RBP42 proteins, allowed us to demonstrate that they are RNA-binding proteins that bind in vitro to a thermodynamically stable ARE element from the 3′ UTR of *HSP70-II* region. Additionally, the steady-state levels of their transcripts were found to be dramatically affected by the temperature of growth, a finding that would suggest a decrease in the de novo protein*-*synthesis of these proteins during heat shock. LbSCD6 and LbRBP42 are cytoplasmic proteins that, under heat-shock conditions, form protein foci around the nucleus. Giving these features regarding RNA-interaction, subcellular localization and heat-shock dependent localization of LbSCD6 and LbRBP42 proteins, a relevant role in mRNA stabilization and RNP storage can be suggested.

## Additional files

Additional file 1: Figure S1. Expression and purification of the recombinant proteins rLbSCD6 (a) and rLbRBP42 (b). Analysis by Coomassie blue staining of their expression in *E. coli*-M15 cells (Lanes 1–3) and purified fractions (Lane 4). Lane 1: total cell extract before induction; Lane 2: total cell extract after induction with IPTG; Lane 3: insoluble fraction of the cell extract; Lane 4: eluted protein after affinity chromatography and in-column refolding procedure. *Abbreviations*: MW, molecular weight marker. (TIFF 673 kb)

Additional file 2: Table S1. Pairwise analysis of amino acids sequence alignment from LbSCD6 and LbRBP42 proteins. The accession number for each sequence is included in Fig. 1 for SCD6 protein and in Fig. 2 for RBP42 protein. (PDF 114 kb)

Additional file 3: Figure S2. Validation of modeled structures for rLbSCD6 and rLbRBP42 proteins. Ramachandran plot for LbSCD6 protein (**a**) and LbRBP42 protein (**b**) were obtained by PROCHECK analysis. (TIFF 1387 kb)

## Abbreviations

ARE: Adenylate/Uridylate-rich element
HSP70: 70-kDa heat-shock protein
RBPs: RNA-binding proteins
SSR: Strand switch regions
