# Supplementary figures and images for: Leishmania braziliensis SCD6 and RBP42 proteins, two factors with RNA binding capacity
Source: Parasit Vectors. 2017 Dec 19;10:610. doi: 10.1186/s13071-017-2557-y (PMC5735676; doi:10.1186/s13071-017-2557-y)

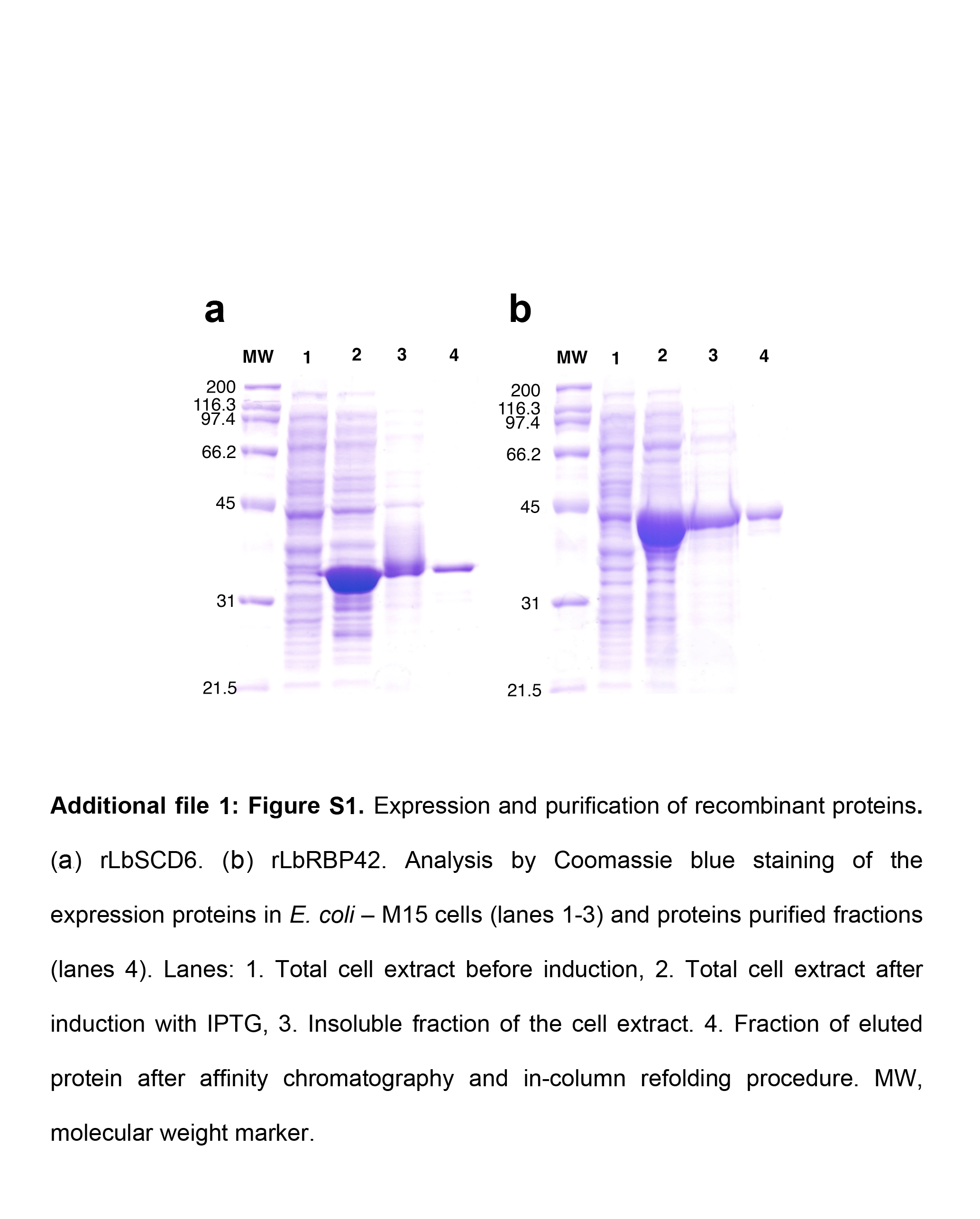

Supplement: Supplementary file 1 — Expression and purification of the recombinant proteins rLbSCD6 (a) and rLbRBP42 (b). Analysis by Coomassie blue staining of their expression in E. coli-M15 cells (Lanes 1–3) and purified fractions (Lane 4). Lane 1: total cell extract before induction; Lane 2: total cell extract after induction with IPTG; Lane 3: insoluble fraction of the cell extract; Lane 4: eluted protein after affinity chromatography and in-column refolding procedure. Abbreviations: MW, molecular weight marker. (TIFF 673 kb) [file 13071_2017_2557_MOESM1_ESM.tif]

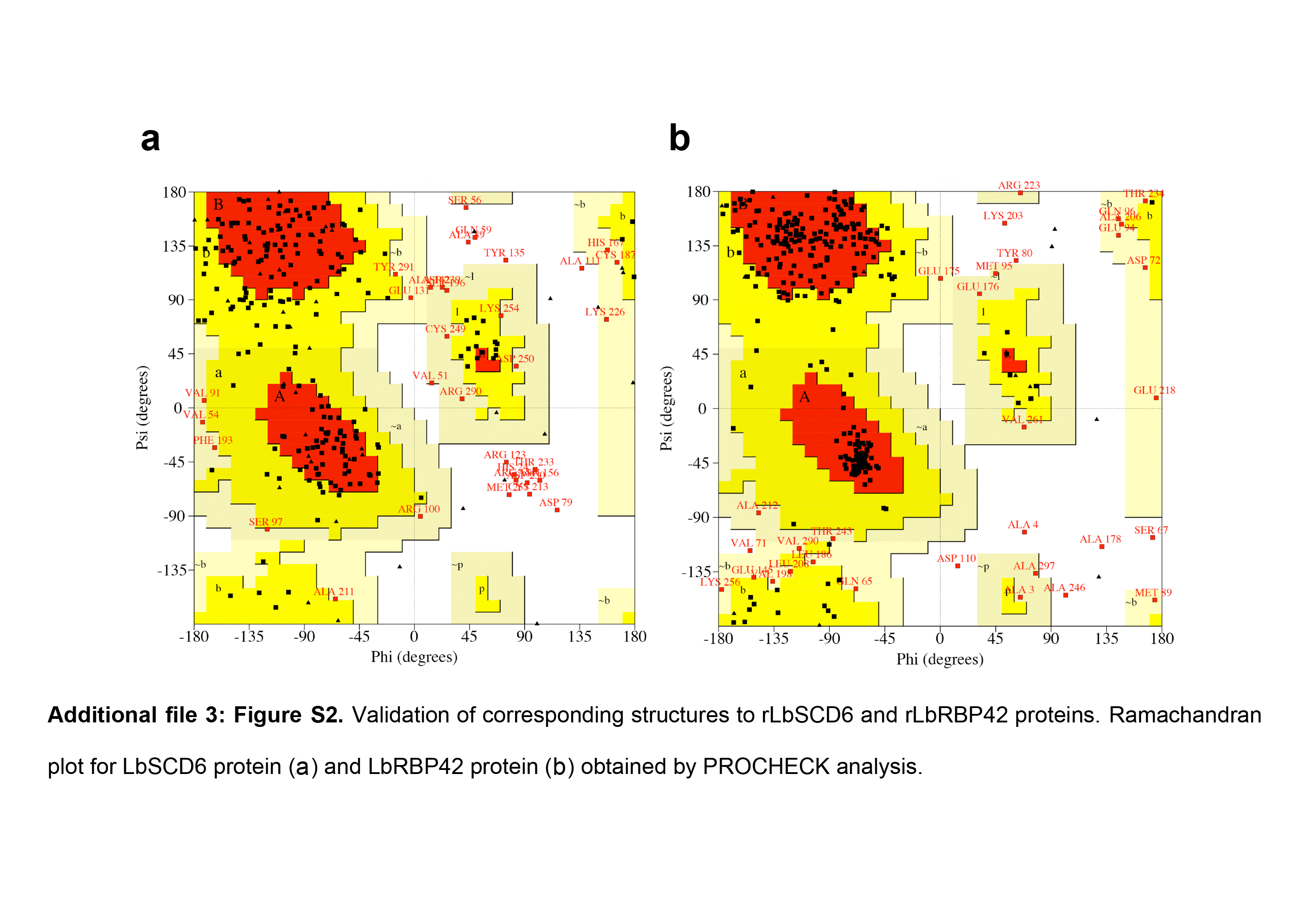

Supplement: Supplementary file 3 — Validation of modeled structures for rLbSCD6 and rLbRBP42 proteins. Ramachandran plot for LbSCD6 protein (a) and LbRBP42 protein (b) were obtained by PROCHECK analysis. (TIFF 1387 kb) [file 13071_2017_2557_MOESM3_ESM.tif]
