# Supplementary material for: Leishmania braziliensis SCD6 and RBP42 proteins, two factors with RNA binding capacity
Source: Parasit Vectors. 2017 Dec 19;10:610. doi: 10.1186/s13071-017-2557-y (PMC5735676; doi:10.1186/s13071-017-2557-y)
Supplement: Supplementary file 2 — Pairwise analysis of amino acids sequence alignment from LbSCD6 and LbRBP42 proteins. The accession number for each sequence is included in Fig. 1 for SCD6 protein and in Fig. 2 for RBP42 protein. (PDF 114 kb) [file 13071_2017_2557_MOESM2_ESM.pdf]

Additional file 2: Table S1. Pairwise analysis of amino acids sequence alignment from LbSCD6 and LbRBP42 proteins. The accession number for each sequence is included in Figure 1 for SCD6 proteins and in Figure 2 for RBP42 proteins.

L. braziliensis SCD6 protein		
	% Identity	% Similarity
L. major	89.2	96.6
T. brucei	48.8	73.7
S. cerevisiae	28.1	56.6
X. leave	27.3	55.2
H. sapiens	28.0	45.7
L. braziliensis RBP42 protein		
	% Identity	% Similarity
L. major	87.3	94.1
T. brucei	34.3	64.5
D. melanogaster	17.4	47.5
H. sapiens	19.9	45.8
